# Soaring Migratory Birds Avoid Wind Farm in the Isthmus of Tehuantepec, Southern Mexico

**DOI:** 10.1371/journal.pone.0092462

**Published:** 2014-03-19

**Authors:** Rafael Villegas-Patraca, Sergio A. Cabrera-Cruz, Leonel Herrera-Alsina

**Affiliations:** 1 Red de Ambiente y Sustentabilidad, Instituto de Ecología A.C., Xalapa, Veracruz, México; 2 Centro de Investigaciones en Ecosistemas, Universidad Nacional Autónoma de México, Morelia, Michoacán, México; University of Western Ontario, Canada

## Abstract

The number of wind farms operating in the Isthmus of Tehuantepec, southern Mexico, has rapidly increased in recent years; yet, this region serves as a major migration route for various soaring birds, including Turkey Vultures (*Cathartes aura*) and Swainson's Hawks (*Buteo swainsoni*). We analyzed the flight trajectories of soaring migrant birds passing the La Venta II wind farm during the two migratory seasons of 2011, to determine whether an avoidance pattern existed or not. We recorded three polar coordinates for the flight path of migrating soaring birds that were detected using marine radar, plotted the flight trajectories and estimated the number of trajectories that intersected the polygon defined by the wind turbines of La Venta II. Finally, we estimated the actual number of intersections per kilometer and compared this value with the null distributions obtained by running 10,000 simulations of our datasets. The observed number of intersections per kilometer fell within or beyond the lower end of the null distributions in the five models proposed for the fall season and in three of the four models proposed for the spring season. Flight trajectories had a non-random distribution around La Venta II, suggesting a strong avoidance pattern during fall and a possible avoidance pattern during spring. We suggest that a nearby ridgeline plays an important role in this pattern, an issue that may be incorporated into strategies to minimize the potential negative impacts of future wind farms on soaring birds. Studies evaluating these issues in the Isthmus of Tehuantepec have not been previously published; hence this work contributes important baseline information about the movement patterns of soaring birds and its relationship to wind farms in the region.

## Introduction

The production of wind energy is increasing rapidly worldwide: in mid- 2013, there was a total of 296 GW of installed capacity, but it was expected to grow for a total of 318 GW for the full year [1]. The Isthmus of Tehuantepec, southern Mexico, is the region with the greatest potential for wind energy yield in the country [2]. It has been estimated that around 2000 MW of wind power could be harnessed in the La Ventosa region alone [3]. Consequently, the 83.3-MW La Venta II wind farm was installed in 2007. By the end of 2012, a total of 15 wind farms were operating in the region, producing 1331.65 MW of energy [4]. However, these installations are located along an important migratory route for raptors that traverses Mexico [5], leading to concerns about the potential impacts of wind farms on birds, as some species of diurnal migrants are commonly observed (particularly during their fall migration), soaring above a ridgeline which in close proximity to La Venta II [6], [7], probably making use of the wind updrafts generated by the walls of the ridge [8].

Birds demonstrate a range of responses to wind farms. For instance, Martínez-Abraín et al. [9] recently suggested that vultures may exhibit a form of behavioural learning to avoid turbines. In comparison, Devereux et al. [10] showed that the positioning of turbines in two wind farms located in East Anglia (England) had no effect on the distribution of some species of wild birds (mostly passerines) occupying agricultural areas. Such observations have led authors to suggest that wind turbines do not represent a serious problem to birds [11], [12]. However, threats that wind farms pose to birds have been catalogued into four main categories: (1) risk of collision, (2) displacement due to disturbance, (3) habitat loss and (4) barrier effect [13], [14], [15].

One form of displacement is when birds adjust their migratory routes (also termed flyways) or local flight paths to avoid wind farms [13]. For instance, it was reported a significant decrease in the number of common eider flocks entering the Nysted offshore wind farm area (Denmark) after the onset of operation [16]. Furthermore, it has been observed that common eiders avoid flying close to or in the area of the Tunø Knob wind park in Denmark [17]. Similarly, Garvin et al. [15] documented a decline in abundance of resident raptor species during the post-construction stage of a wind farm in Wisconsin (USA). de Lucas et al. [18] showed that soaring birds (e.g. Griffon Vulture *Gyps fulvus*, Black Kite *Milvus migrans* and White Stork *Ciconia ciconia*) detect and avoid the presence of wind turbines of a wind farm in Tarifa (Spain) better when these were functioning. However, more research is needed because bird species exhibit a range of responses to wind-energy facilities, with other factors, such as site and season, also playing a role. Specific studies are necessary to assess the response of birds to different types of wind farms in different locations.

Hence, in the current study, we documented the flight trajectories of soaring birds in the vicinity of the La Venta II wind farm in southern Mexico, during both the spring (northward passage) and fall (southward passage) migratory seasons of 2011. This work provides preliminary insights about the potential relationship between the flight trajectories of migrating soaring birds and the topography surrounding a wind farm.

## Materials and Methods

### Study Area

**Figure 1:**
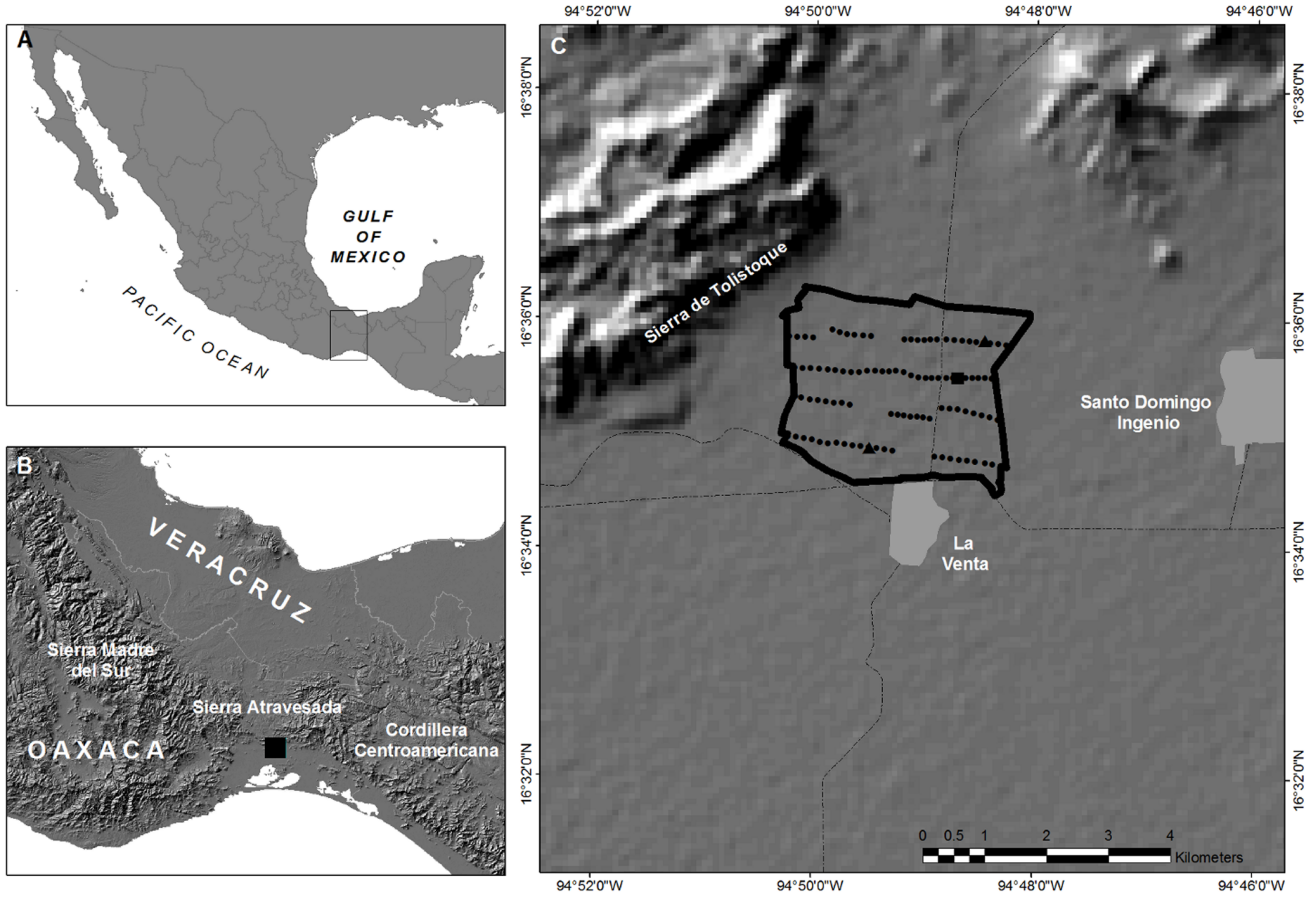
Study area. (a) Isthmus of Tehuantepec in southern Mexico, (b) location of the La Venta II wind farm in Oaxaca, (c) arrangement of wind turbines, surrounding topography, and nearby towns. Black square within the wind farm (c) shows the location of the radar monitoring station in both seasons; black triangles show the location of the hawk-watch stations in fall (∼700 m northeast of radar) and spring (∼2 km southwest).

The La Venta II wind farm is located less than 1 km north of Ejido La Venta, which is a small town on the Pacific slope of the Isthmus of Tehuantepec, a narrow region that separates the Gulf of Mexico from the Pacific Ocean (Fig. 1). The Isthmus is an important corridor for migratory birds moving between North and South America [19] and an important stopover site for migratory birds in the fall [20]. During construction of the wind farm, the area was described as a world-class bird migration corridor [21]. The La Venta II wind farm has 98 turbines arranged in four rows that are aligned from west to east, with a total nominal capacity of 83.3 MW distributed in a 9.49-km^2^ area. The farm is located on the inland edge of the Pacific coastal plain. The wind facility is quite close to an orographic (mountain chain) system. Specifically, it is located less than 2 km from the southeast tip of the Sierra de Tolistoque range, which is a small ridgeline with a maximum altitude of 700 m above sea level (ASL) running in a west-east direction. In addition, the wind facility is located ∼3 km southwest of some low-altitude hills and light slopes [7].

### Radar Equipment

We used an X band marine radar (Model FR-1525 Mark 3, Furuno, Nishinomiya, Japan) mounted on a truck adapted to serve as a mobile unit. Similar radar laboratories have been described by Cooper et al. [22] and Harmata et al. [23]. The radar transmitted at a frequency of 9,140 MHz through a 2-m-long slotted waveguide (antenna), with a maximum output of 25 kW and was operated with a pulse length of 0.07 μs. The display unit had a range resolution of 35 m. The antenna emitted a beam with a width of 1.23° (horizontal) ×20° (vertical), with side lobes ±10° [22]. The unit was powered with a low-noise electric generator.

### Study Design and Data Collection

We observed the movements of soaring birds during two migratory seasons (spring and autumn of 2011) using a single marine radar within the wind farm, sited on the service road of the northern-most row of turbines (16.596992 °N latitude, −94.811981°W longitude, 14 m ASL) where the surrounding vegetation served as a radar fence. Observations started at approximately 09:00 and included 4 to 6 continuous 1-h sampling sessions per day for 13 days in spring (between March 31^st^ and April 29^th^) and for 15 days in fall (between October 5^th^ and 25^th^). These dates and times of the day coincided with the known peak of diurnal migratory activity of raptors in the vicinity of the La Venta II wind farm (Villegas-Patraca, unpublished data). Each hourly session was subdivided into: 1) 10 min to adjust the radar, 2) 20 min to observe and collect data on the flight trajectories, 3) a 10-min break and 4) 20 min to continue collecting data.

From the radar display, we recorded the flight directions and three sets of polar coordinates for every trajectory (i.e., the start-, end- and mid-points), which were measured with a compass and index line from the screen. All data were recorded manually onto a laptop computer. We used the term “target” to designate objects detected by the radar because it did not allow unequivocal identification. However, based on concurrent direct observations from a hawk-watch monitoring station in both seasons (Fig. 1), we confirmed that most of the detected targets on the radar were either individuals or flocks of soaring birds. The radar was operated in surveillance mode, with a 6-km detection radius. This setting has been proven useful for the detection of soaring birds when using this type of radar [22] (and personal observations). We did not measure flight altitudes. Data is available at http://dx.doi.org/10.6084/m9.figshare.938235.

Our study did not involve handling bird specimens. The only permit needed was to access the wind farm, which was kindly provided by CFE (Comisión Federal de Electricidad). No further permits were required for the described monitoring.

### Data Analysis

We loaded the polar coordinates into R 2.15.1 [24], on which the flight trajectories of each season were plotted by joining their start-, mid- and end-points. We also plotted the polygon showing the perimeter of the wind turbines of La Venta II wind farm and then estimated how many times this polygon was intersected by the documented flight trajectories. Then, we ran simulations of the documented flight trajectories 10,000 times under different scenarios or null models for each season (Table 1). To obtain an index that was comparable between the real and simulated data, we divided the number of intersections by the total length of all trajectories (in km) and, finally, constructed a frequency distribution of the intersections/km for both the observed and simulated trajectories. If the observed number of intersections/km fell in the 250 smallest or largest values of the distribution from the simulated data, the hypothesis of randomness was rejected at α = 0.05. We analysed the directions of trajectories with the circular statistics software Oriana ver. 4.01 [25], reporting the mean flight direction (*µ*) and the length of the main vector (*r*). We also report the species of soaring birds observed from the hawk-watch monitoring station, and their abundances.

**Table 1:** Different models or scenarios used to simulate the flight trajectories.

Model	Restrictions
1	None. Start-, mid-, and end-points were randomly generated to obtain the same number of trajectories as we observed in each season, resulting in completely random trajectories within the detection radius, and in a very broad model
2	We retained the observed start- points, randomly generating the mid- and end-points. This scenario simulated trajectories that followed new paths (no restriction of direction and length), but started from the same start- points as the observed ones.
3	We retained both the observed start- and mid-points, and randomly generated the end-points. This scenario simulated alternative trajectories, after the “simulated flocks” had passed through the observed start- and mid-points. We did not restrict the length or the direction of the trajectory between the observed mid- and simulated end-points
4	We retained the observed start- and end- points; however, the mid-point was randomly generated in the rectangular space formed by the intersection of imaginary lines extending from the x- and y-axis of the start- and end-points. This scenario simulated alternative trajectories between the observed start- and end-points
5	We only applied this model to the fall season dataset. We retained both the observed start- and mid-points, randomly generating the end-points south of the former two. This scenario considered the seasonal tendency of flight directions, simulating alternative endings of the trajectories after the “simulated flocks” had passed through the observed start- and mid-points. We did not restrict the length of the trajectory between the observed mid- and simulated end-points. We did not apply this model to the spring data because flight directions did not show a marked pattern.

## Results

### Fall

**Figure 2:**
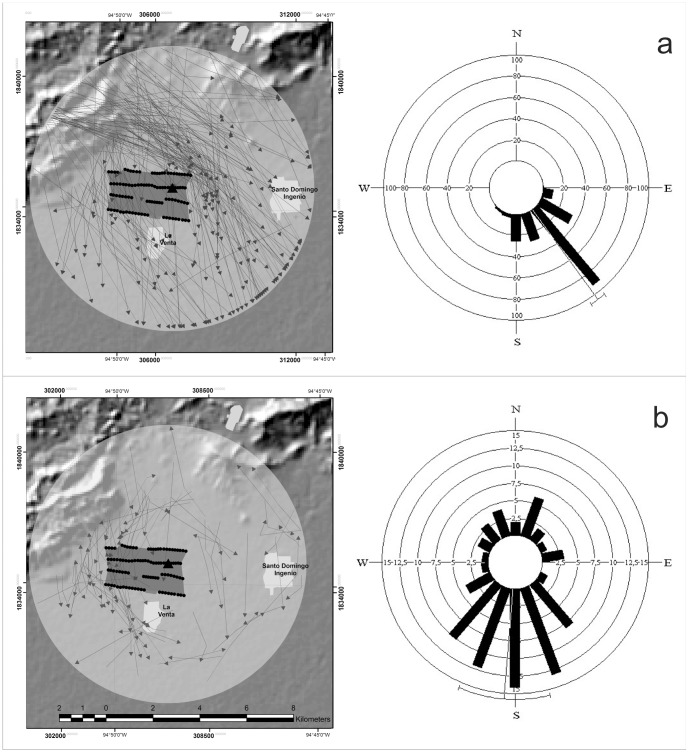
Flight trajectories and directions. Each line in left panels represent the flight trajectory of one flock, black triangles at the centre shows the location of the radar monitoring station. Left panels represent a summary of flight directions. Upper panels (a) autumn, lower panels (b) spring.

During the fall season, we recorded 193 flight trajectories, with a total length of 1,447.68 km. The mean flight direction (*μ*) was 143.9°, with the observed trajectories being closely clustered around the mean (*r* = 0.91), supporting the expected flight direction for fall, when Nearctic-Neotropical migrants fly south to their wintering grounds (Fig. 2). The most abundant soaring bird species identified by the hawk-watch station was the Turkey Vulture *C. aura* (n = 266,977), followed by Swaninson's Hawk *B. swainsoni* (n = 66,545); hence, we assumed that most of the trajectories were from flocks of these species, though the hawk-watch station identified several others (Table 2). During the autumn season, the polygon defined by the rows of wind turbines was intersected 90 times by the flight trajectories, resulting in a total of 0.0621 intersections/km. This index was lower than that obtained under the five simulated scenarios or null models (Table 3).

**Table 2:** Species and abundances of soaring birds identified from the hawk-watch monitoring station.

Spring		Fall	
Species	No.	Species	No.
*Leucophaeus pipixcan*	13402	*Cathartes aura*	266977
*Cathartes aura*	1286	*Buteo swainsoni*	66545
*Coragyps atratus*	1174	*Buteo platypterus*	8685
*Mycteria americana*	59	*Pelecanus erythrorhynchos*	1701
*Caracara cheriway*	36	*Mycteria americana*	875
*Buteo albicaudatus*	26	*Falco sparverius*	119
*Buteo swainsoni*	19	*Coragyps atratus*	95
*Buteo magnirostris*	11	*Accipiter sp.*	38
*Fregata magnificens*	9	*Caracara cheriway*	20
*Falco sparverius*	7	*Buteo albicaudatus*	16
*Buteo platypterus*	4	*Pelecaniforme*	14
*Buteo nitidus*	3	*Circus cyaneus*	13
*Buteo brachyurus*	2	*Fregata magnificens*	12
*Circus cyaneus*	1	*Accipiter striatus*	9
*Falco columbarius*	1	*Buteo jamaicensis*	6
*Falco peregrinus*	1	*Accipiter cooperii*	4
*Pandion haliaetus*	1	*Buteo nitidus*	4
		*Falco femoralis*	4
		*Falco peregrinus*	3
		*Falco sp.*	3
		*Buteo brachyurus*	2
		*Buteo albonotatus*	1
		*Buteo magnirostris*	1
		*Elanus leucurus*	1
		*Pandion haliaetus*	1

**Table 3:** Summary of the null model results.

	Intersections/km					
	Observed	Model 1	Model 2	Model 3	Model 4	Model 5
**Spring**	0,108	**0,147–0,201**	**0,145–0,196**	**0,142–0,193**	0,098–0,142	-----*
**Fall**	0,062	**0,156–0,193**	**0,140–0,173**	**0,155–0,141**	**0,091–0,116**	**0,132–0,157**

Ranges represent 95% of the number of intersections/km from 10,000 trajectories under the simulated scenarios or null models for the spring and fall seasons. Bold type indicates ranges that do not include the observed number of intersections/km (p<0.05).

*Not evaluated.

### Spring

During the spring season, we recorded 87 trajectories, with a total length of 257.47 km. The mean flight direction was 184.8° (*μ*) which differed to the expected flight direction for spring, when Nearctic-Neotropical migrants fly back to their breeding grounds in North America; however, the observed trajectories were widely scattered, showing a low concentration around the mean (*r* = 0.42, Fig. 2). The most abundant soaring bird species identified at the hawk-watch station was the Franklin's gull *Leucophaeus pipixcan*, followed by the Turkey Vulture *C. aura* and Black Vulture *Coragyps atratus*, (n = 13,402, 1286 and 1174 respectively; Table 2). During the spring season, the polygon representing the wind turbines was intersected 28 times by the flight trajectories, resulting in a total of 0.1087 intersections/km. This index was lower than that obtained under Models 1, 2 and 3, leading us to reject the hypothesis that the number of observed intersections was random under these scenarios. In comparison, Model 4 did not depart from the null distribution (Table 3).

## Discussion

Different responses by birds to the presence of wind farms have been observed around the world [9], [10], [15], [26]. Here we present the first report of potential avoidance behaviour for a Mexican wind farm. La Venta II was inaugurated in 2007 [27], becoming the first large operational wind farm in Mexico. However, there has been a rapid increase in the number of wind energy developments in the region, which is part of one of the most important bird migration routes in North America [5] and is known to support large numbers of Swainson's Hawks *B. swainsoni* and Turkey Vultures *C. aura* during the fall migratory season [28], [29], whereas the most common species of soaring bird in spring is the Franklin's Gull *L. pipixcan*.

Avoidance behaviour has been observed in coastal [26], inland [15], [18] and offshore [16], [30] wind farms. But many factors influence whether birds avoid or enter wind energy facilities [31], [32]. Hence, research at local-scales is required to evaluate how birds respond to site-specific conditions. Our goal was to provide the first documented accounts of how soaring birds responded to a wind farm in the Isthmus of Tehuantepec. In spring and fall, the observed number of intersections/km fell outside of the null distributions obtained under Models 1, 2 and 3. Even the completely random simulated trajectories intersected the wind farm more often than the real observed flight trajectories. This difference between expected and observed trajectories indicates that birds were exhibiting an avoidance pattern of movement. The same conclusion was obtained from Models 4 and 5, which had a highly restricted design, during the fall season. In these two models, our results again showed a lower rate of actual intersections/km compared to that obtained under the simulated scenarios. Conversely, in spring, Model 4 did not depart from the null distribution. In this instance, our results indicated that the observed number of intersections/km might be random, with no pattern of avoidance. Although our results indicate that migrating soaring birds avoided La Venta II, we lack of comparable data from the pre-construction stage, hence we cannot really assess if the observed patterns are a response to the presence of the wind farm. However, a 5-days survey made during one fall season before La Venta II was built, suggests a similar pattern of flight trajectories as reported in this study [6].

The pattern observed in fall might be explained by geographical features. For instance, the Sierra de Tolistoque is a ridgeline located to the northwest of the wind farm, where the interaction of the wind with its walls may generate valuable resources (updrafts) which may provide suitable airspace habitat [33] temporarily used for continued soaring flights. Flocks of raptors are commonly observed flying above the ridge during fall [6], [7], and most of the flight trajectories recorded during fall in this study started from this location. Given the position and (west-east) orientation of the Sierra de Tolistoque, it is possible that the observed flight trajectories of soaring birds in fall did not intersect La Venta II because the ridgeline naturally guided the birds away from the farm. In spring, soaring birds are expected to approach the wind farm from the south, where a close prominent ridge is not available to lead the direction of birds. In that season we recorded less flight trajectories, but most of them were clustered on the west side of La Venta II, close to Sierra de Tolistoque, suggesting again that this ridgeline may be playing an important role in the observed pattern, probably as a landmark used by soaring birds to guide their journeys.

Our results suggest that La Venta II do not represent a serious threat to migrating soaring birds, hence we consider it to have a fortunate location, as it was decided considering mainly the availability of the wind resource [6]. Other studies have obtained similar conclusions, leading some authors to suggest that wind farms do not represent a substantial risk to birds [12]. However, we need to warn that the observed pattern might be site-specific to La Venta II, with the surrounding geographical features playing a significant role. Therefore, similar studies are urgently required at the other wind farms located on the Isthmus, as all are sited on the same migratory route. This requirement is particularly important, as previous studies have shown that raptors behave differently at different sites, even when in close proximity [26], and because the Mexican government aims to increase the production of clean energy during the next years, potentially including the installation of >200 wind farms in the country [34].

Although it has been suggested that collision-related fatalities do not have an effect of populations of birds [35], a recent study estimates that a mean of 234,000 birds are killed annually by collisions with wind turbines in the contiguous United States alone [36]. Hence, although our results show that La Venta II represents a low risk to migrant soaring birds, further and continued studies are necessary considering the potential cumulative impacts that several wind farms clustered on the Isthmus might have on migrant birds; besides, La Venta II has an expected useful life of ∼20 years [6], which might be similar for other nearby wind farms. This and the above mentioned plans for future energy production in Mexico, suggest that wind farms in the Isthmus are not to be removed from the landscape in the close future, but the opposite. Furthermore, the effects of La Venta II on resident bird species should also be evaluated. Such work is important, because it has been suggested that this particular wind farm may become a local population sink for resident species such as the White-tailed Hawk *Buteo albicaudatus*, as some carcasses of this species have been found within this wind farm [21], and because we have found more carcasses of resident than of migratory species (unpublished data).

Although our study may be technically simple, our results advance existing knowledge about how soaring birds respond to wind farms in this particular area, which is highly used by some species of migrant soaring birds. Furthermore, we suggest that geographical features play a potentially important role in aiding soaring birds to avoid wind farms, an issue that may be considered by decision-makers and wind-energy developers in the region as part of their strategies to minimize the negative impacts of future wind farms on birds. However, we also highlight the need for continued and improved studies in the Isthmus, to raise the much needed information for the region.
